# The impact of Bacillus Calmette-Guérin vaccination on antibody response after COVID-19 vaccination

**DOI:** 10.1016/j.isci.2023.108062

**Published:** 2023-09-28

**Authors:** Esther J.M. Taks, Simone J.C.F.M. Moorlag, Konstantin Föhse, Elles Simonetti, Christa E. van der Gaast-de Jongh, Cornelis H. van Werkhoven, Marc J.M. Bonten, Jaap ten Oever, Marien I. de Jonge, Janneke H.H.M. van de Wijgert, Mihai G. Netea

**Affiliations:** 1 Department of Internal Medicine, Radboud University Medical Center, Nijmegen, the Netherlands; 2 Radboud Center for Infectious Diseases, Radboud University Medical Center, Nijmegen, the Netherlands; 3 Department of Laboratory Medicine, Laboratory of Medical Immunology, Radboud University Medical Center, Nijmegen, the Netherlands; 4 Julius Center for Health Sciences and Primary Care, University Medical Center Utrecht, Utrecht, the Netherlands; 5 Department for Immunology and Metabolism, Life and Medical Sciences Institute (LIMES), University of Bonn, Germany

**Keywords:** health sciences, medical science, microbiology, virology

## Abstract

Earlier studies showed that BCG vaccination improves antibody responses of subsequent vaccinations. Similarly, in older volunteers we found an increased IgG receptor-binding domain (RBD) concentration after SARS-CoV-2 infection if they were recently vaccinated with BCG. This study aims to assess the effect of BCG on the serum antibody concentrations induced by COVID-19 vaccination in a population of adults older than 60 years. Serum was collected from 1,555 participants of the BCG-CORONA-ELDERLY trial a year after BCG or placebo, and we analyzed the anti-SARS-CoV-2 antibody concentrations using a fluorescent-microsphere-based multiplex immunoassay. Individuals who received the full primary COVID-19 vaccination series before serum collection and did not test positive for SARS-CoV-2 between inclusion and serum collection were included in analyses (n = 945). We found that BCG vaccination before first COVID-19 vaccine (median 347 days [IQR 329–359]) did not significantly impact the IgG RBD concentration after COVID-19 vaccination in an older European population.

## Highlights

• BCG vaccine prior to COVID-19 vaccines does not increase anti-SARS-CoV-2 antibodies
• Sex differences in antibody concentration are still present in older adults
• Interval between BCG and subsequent COVID-19 vaccination may play a role in its effect

## Graphical abstract

**Figure:**
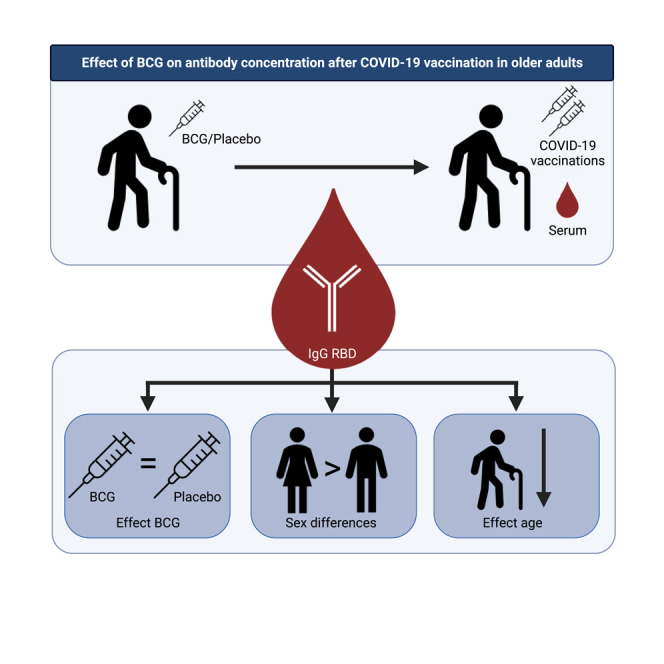

## Introduction

During the coronavirus disease 2019 (COVID-19) pandemic, several approaches have been investigated to boost the immune response of those vulnerable for severe COVID-19. The Bacillus Calmette-Guérin (BCG) vaccine, which is commonly used to protect against tuberculosis, was considered as a potential measure to boost host defense mechanisms before specific COVID-19 vaccines became available.^1^^,^^2^^,^^3^^,^^4^ BCG vaccination induces trained immunity, which is functional reprogramming of innate immune cells providing heterologous protection.^5^ BCG has been shown to have a protective effect against several viral infections including influenza^6^ and reduced viraemia after live-attenuated yellow fever vaccination as model for human viral infection.^7^ On the other hand, the effects of BCG on COVID-19 incidence and severity are mixed at best, with the largest studies in both healthcare workers and the elderly failing to show a reduction in susceptibility,^8^^,^^9^^,^^10^ although an effect on disease severity, including hospitalization and death, cannot be excluded.^11^

Older adults are at risk for developing severe COVID-19 and also less able to produce an adequate adaptive immune response upon vaccination.^12^ Antibody responses in older adults are reduced compared with younger adults, likely due to immune senescence, which includes a decline in naive B cells.^13^ In addition, the antibodies produced are also of lower affinity.^13^

Previous studies have shown an improved serological response against influenza vaccination in individuals recently vaccinated with BCG.^14^ Even though the clinical results are mixed, several small studies have found that recent BCG revaccination, 1–12 months prior to COVID-19 vaccination, improves the antibody response induced by specific SARS-CoV-2 vaccines.^15^^,^^16^ In the current cohort, we previously found in a relatively small group (n = 30) that compared with placebo, recent BCG vaccination led to an improved IgG-receptor-binding domain (RBD) antibody concentration in older individuals 2–6 months after COVID-19 infection.^8^ During this study, COVID-19 vaccination programs started, and many participants received the primary vaccination series during follow-up.

Considering these non-specific effects of BCG and the importance to identify such an effect in the elderly, we aim to determine the effect of BCG vaccination compared with placebo vaccination on COVID-19-vaccination-induced SARS-CoV-2 antibody concentration in a Western European population.

## Results

### Participant characteristics

**Figure 1:**
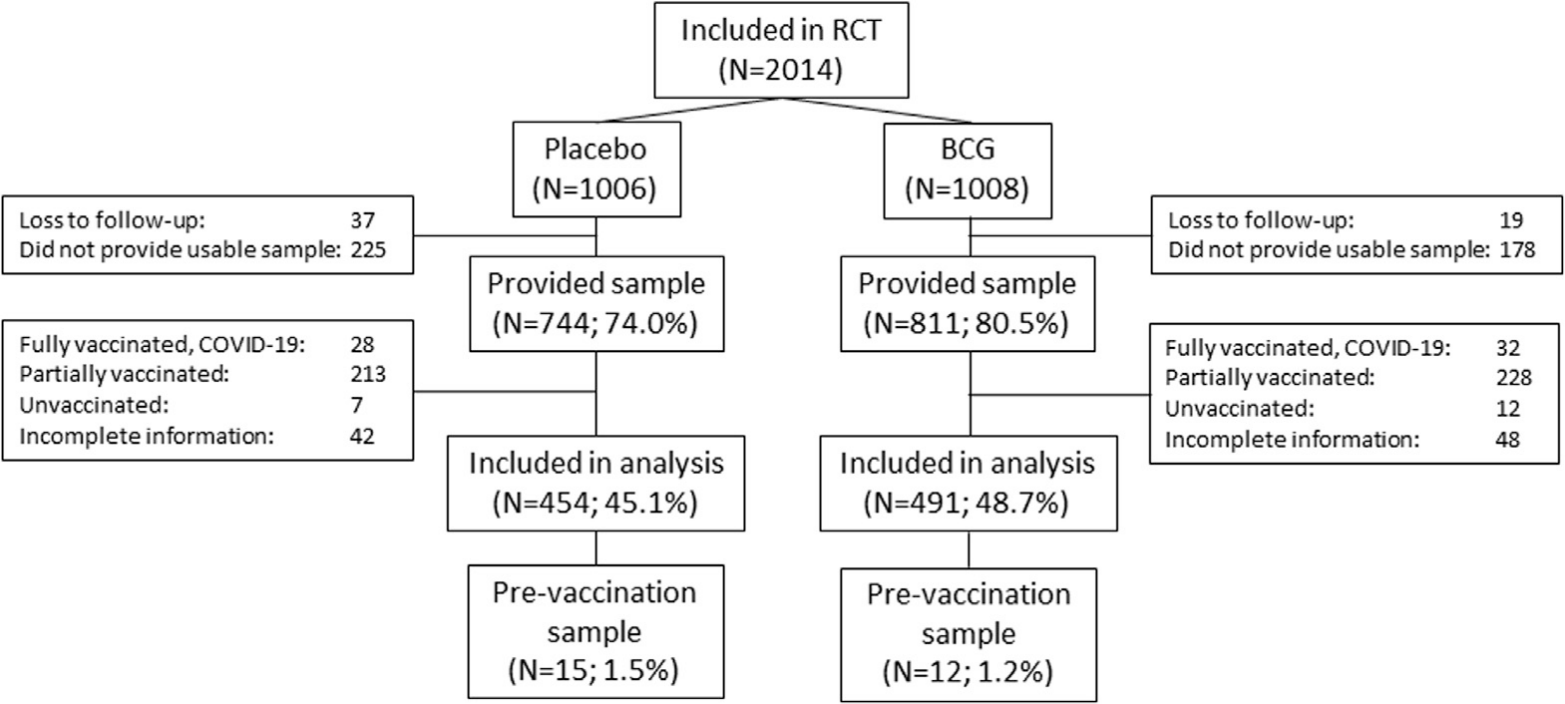
CONSORT diagram Flow diagram showing the participants included in this analysis of antibodies from the BCG-CORONA-ELDERLY cohort.

One thousand five hundred fifty-five of the 2014 participants (77.2%) who were included in the BCG-CORONA-ELDERLY trial returned the serum tube for serology testing. At the time of sampling, most of the participants who provided complete COVID-19 vaccination history reportedly received at least one specific COVID-19 vaccine (n = 1448; 98.8% of the samples). Of the 1,555 participants, 1,005 were fully vaccinated against SARS-CoV-2, 441 were partially vaccinated, 19 reported not having received any vaccine, and 90 people provided us with no or incomplete information about their COVID-19 vaccination status. Sixty of the fully vaccinated participants had experienced a PCR-confirmed SARS-CoV-2 infection between inclusion and serum collection and were excluded from analysis, leaving 945 fully vaccinated individuals for the analysis (Figure 1). Reasons for exclusion were comparable between the BCG and placebo group.

Most of the 945 participants analyzed were aged 60–69 years (n = 496; 52.5%), with only few adults aged 80+ years (n = 66; 7.0%); 49.4% of participants were female (n = 467). BNT162b2 was the most frequently administered vaccine in this group (82.9%), followed by ChAdOx1-S/nCoV-19 (15.6%), mRNA-1273 (1.4%), and Ad26.COV.S (0.2%). There were no large differences in baseline characteristics between placebo and BCG groups; however, there was a difference in sex distribution, with 53.5% men in the placebo group and 47% in the BCG group (p = 0.054 by chi-squared test) (Table 1); 454 out of 1,006 (45.1%) randomized to placebo and 491 out of 1,008 (48.7%) randomized to BCG vaccination were part of the analysis population.

**Table 1:** Baseline and COVID-19 vaccination characteristics of participants

Variable	Placebo n = 454	BCG n = 491
Median age (IQR)—yr	69 (65–73)	69 (65–73)
Age category—no. (%)		
60–69	234 (51.5)	262 (53.4)
70–79	193 (42.5)	190 (38.7)
80+	27 (5.9)	39 (7.9)
Sex—no. (%)		
Male sex	245 (54.0)	233 (47.5)
Female sex	209 (46.0)	258 (52.5)
Median BMI (IQR)—kg/m^2^	24.8 (23.0–27.5)	24.9 (23.0–27.5)
Comorbidities—no. (%)		
Diabetes mellitus	29 (6.4)	31 (6.3)
Hypertension	151 (33.3)	155 (31.6)
Cardiovascular disease	93 (20.5)	91 (18.5)
Chronic kidney disease	8 (1.8)	8 (1.6)
Asthma	26 (5.7)	27 (5.5)
Vaccination—no. (%)		
BNT162b2	377 (83.0)	406 (82.7)
ChAdOx1-S/nCoV-19	71 (15.6)	76 (15.5)
mRNA-1273	6 (1.3)	7 (1.4)
Ad26.COV.S	0 (0)	2 (0.4)
Median time between last vaccine and serology (IQR) —days	34 (24–52)	35 (24–54)
BNT162b2	35 (25–54)	36 (26–59)
ChAdOx1-S/nCoV-19	25 (18–37)	25 (18–32)
mRNA-1273	47 (45–54)	48 (37–113)
Ad26.COV.S	n.a.	52 (46–57)
Median time between BCG/placebo and first COVID-19 vaccine (IQR)—days	348 (334–359)	347 (329–359)
BNT162b2	351 (337–360)	351 (334–361)
ChAdOx1-S/nCoV-19	334 (316–345)	327 (309–337)
mRNA-1273	334 (322–337)	339 (308–345)
Ad26.COV.S	n.a.	351 (349–352)

The interval between last SARS-CoV-2 dose and sample collection in the included population for all vaccines was 14–159 days with a median of 34 days (IQR 24–52.5), which was similar for both groups (BCG 35 days (IQR 24–54), and placebo 34 days (IQR 24–52) (Table 1).

Baseline characteristics of those not included in the analysis can be found in Table S1. The analyzed population is older than those that were excluded from this analysis (median age is 69 years in the analyzed population compared with 66 years). In the Netherlands, there was an age-specific COVID-19 vaccination strategy, which meant that the oldest individuals were considered most at risk and vaccinated first. In addition, ChAdOx1-S/nCoV-19 was available to those aged between 60 and 69 years and required a larger interval between the two vaccines; therefore, many individuals in this age category were only partially vaccinated. Both factors might contribute to the difference in age category between the included and excluded population.

### No increased IgG RBD antibody concentration post-COVID-19 vaccination in those who received the BCG vaccine

**Figure 2:**
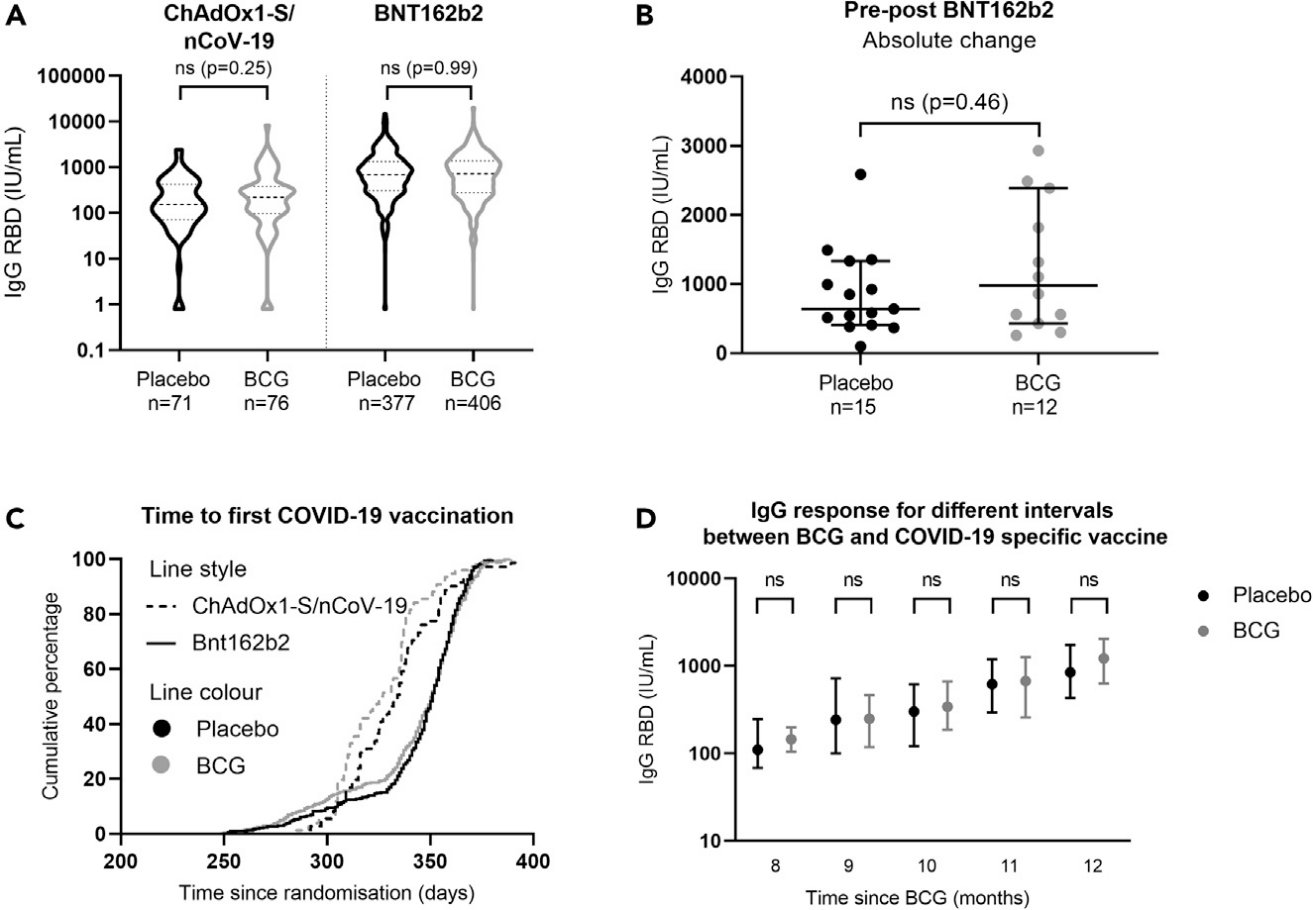
The effect of BCG on the antibody response in older adults. The concentrations of immunoglobulin G against the receptor-binding domain (RBD) on the spike protein in international units per milliliter (IU/mL) comparing BCG vaccination (gray) with placebo (black) (A) Violin plot showing the effect of BCG after full COVID-19 vaccination with ChAdOx1-S/nCoV-19 or BNT162b2. A Mann-Whitney U test was performed to compute differences between those who had received BCG and placebo, the dashed line represents the median, the dotted line is the interquartile range (IQR). (B) Comparison of absolute change in IgG against RBD in individuals before and after BNT162B2 vaccination using a Mann-Whitney U test, with bars showing the median and IQR. (C) Survival curve showing the time to first vaccination for the different vaccine types (line style) by treatment arm (line color) with the cumulative percentage of participants who had received their first COVID-19 vaccine on the y axis. Differences were computed using a log rank test. (D) Comparison of median IgG RBD concentration and IQR based on the interval between BCG vaccination and first COVID-19 vaccine by month analyzed by Mann-Whitney U test. ‘ns’ is used to indicate that there is no statistically significant difference between the groups analyzed.

For ChadOx1-S/nCoV-19 vaccination, those who had received placebo (n = 71) had a median IgG RBD concentration of 153.4 IU/mL (IQR 71.4–422.6) compared with 220.5 IU/mL (IQR 96.6–380.7) in the BCG-vaccinated group (n = 76). For BNT162b2 vaccination, IgG RBD concentration was 689.1 IU/mL (IQR 312.2–1332.0; n = 377) and 721.6 IU/mL (IQR 276.8–1388; n = 406), respectively. Although the median is slightly higher in the BCG-vaccinated group, differences in IgG RBD antibody concentration in individuals previously vaccinated with BCG compared with placebo were not statistically significant (Figure 2A).

From a small group of participants (n = 27), there was also EDTA plasma available from approximately 4 months before serum collection, when they had not been immunized against SARS-CoV-2 yet. After this small group had been fully vaccinated, all with Bnt162b2, there was no significant difference in the absolute change of IgG RBD between those who received a placebo (n = 15) with a median of 641.1 IU/mL (IQR 409.3–1335), compared with those BCG vaccinated (n = 12) with a median of 982.2 IU/mL (IQR 465.0–2245.0) one year earlier (Figure 2B).

Time to first COVID-19 vaccination since randomization was similar for the compared groups (Figure 2C). Median time to vaccination for those vaccinated with ChadOx1-S/nCoV-19 was 334 days (IQR 316–345) in the placebo group and 327 days (IQR 309–337) in the BCG group (p = 0.06). The first vaccination with BNT162b2 took place after a median of 351 days in both placebo and BCG-vaccinated groups (p = 0.38). Stratified for different intervals between BCG/placebo vaccination and COVID-19 vaccination, there were also no statistically significant differences in IgG RBD between the BCG and placebo group (Figure 2D; Table S2).

Full information on SARS-CoV-2 antibody concentrations from those in the BCG-CORONA-ELDERLY who provided full COVID-19 vaccination history regardless of COVID-19 infection and number of vaccines can be found in Table S3.

### Women have an increased antibody response irrespective of BCG status

**Figure 3:**
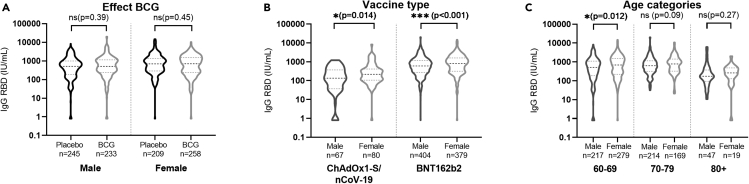
Sex differences in antibody responses in older adults (A) The difference in IgG RBD between men and women by vaccine using a Mann-Whitney U test. (B) The difference in IgG RBD between men and women by age category. (C) The difference in IgG RBD separated for men and women by age category. For all panels data are represented as median (dashed line) +/− IQR (dotted line). Asterisks indicate statistical significance (∗p < 0.05, ∗∗p < 0.01, ∗∗∗p < 0.001).

Within the specific sex there was no significant difference in IgG RBD levels between the BCG and placebo group (Figure 3A). However, irrespective of BCG vaccination, we found significant higher median IgG RBD antibody concentration in women compared with men for both ChAdOx1-S/nCoV-19 (median 218.7 IU/mL [IQR 106.8–413.8] in women and 134.6 IU/mL [IQR 43.2–365.6] in men [p = 0.014]) and BNT162b2 vaccine (median 819.2 IU/mL [IQR 319.7–1587.8] in women versus 600.7 IU/mL [277.3–1147.9] in men [p = 0.001]) (Figure 3B). The difference only reached statistical significance in the participants aged 60–69 years (median 698.3 IU/mL [IQR 239.0–1526.2] in women compared with 515.3 IU/mL [IQR 184.4–1091.4] in men [p = 0.011]) (Figure 3C). In the older age groups the differences were non-significant and, however, showed the same direction (an advantage for women) as in the 60–69 age category. Differences in statistical power may account for this difference, especially in the oldest category.

### The impact of age on the humoral immune response

IgG RBD antibody concentrations were not correlated with age when all COVID-19 vaccinations were combined (p = 0.09, r(945) = 0.05 by Spearman rank, 95% confidence interval [CI] −0.01; 0.12) irrespective of BCG or placebo vaccination. In those BCG-vaccinated, there was no significant inverse correlation between age and IgG RBD (p = 0.83, r(491) = 0.01 [95% CI −0.08; 0.10]), and although statistically significant (p = 0.03) within the placebo group there was a negligible positive correlation between age and IgG RBD (r(454) = 0.10 [95% CI 0.01; 0.19]).

**Figure 4:**
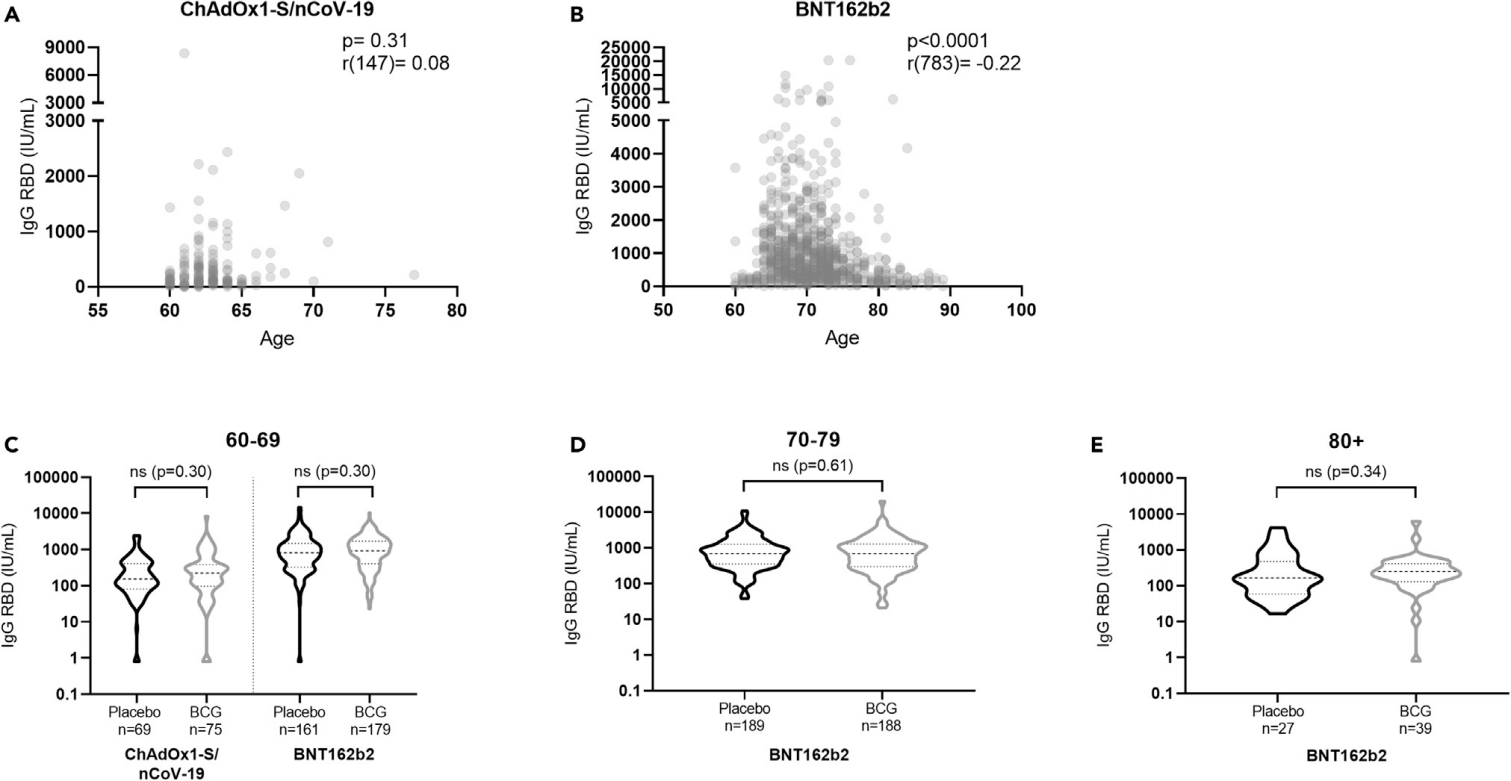
The correlation between age and antibody response (A) Spearman’s rank correlation between age and IgG RBD in those fully vaccinated with ChAdOx1-S/nCoV-19. (B) Spearman’s rank correlation between age and IgG RBD in those fully vaccinated with the BNT162b2 vaccine. Due to the large range in IgG RBD concentration for the different vaccines the y axis of 4a and 4b are different. (C–E) Mann-Whitney U test on differences between BCG- and placebo-vaccinated individuals after BNT162b2 vaccination separated by age category. Data are represented as median (dashed line) +/− IQR (dotted line). ‘ns’ is used to indicate that there is no statistically significant difference between the groups analyzed.

In the ChadOx1-S/nCov-19-vaccinated group, there was no correlation between age and antibody response (p = 0.31 with r(147) = 0.08, 95% CI −0.08; 0.25) (Figure 4A), whereas there was a significant correlation (p < 0.0001 with r(783) = −0.22, 95%CI −0.29; −0.15) in those vaccinated with BNT162b2 (Figure 4B). However, differences in statistical power and a limited age category who was given the ChadOx1-S/nCov-19 vaccine may explain differences between the vaccines.

When assessing the separate vaccines, we also found no significant differences by Mann-Whitney U test between those placebo and BCG-vaccinated per age category. In age category 60–69 years, both ChadOx1-S/nCov-19 and BNT162b2 were available but not significantly different (median 143.1 IU/mL [IQR 71.4–410.8] in placebo versus 222.7 IU/mL [IQR 98.7–378.5] and BNT162b2 [median 811.9 IU/mL [IQR 326.4–1492.6] in placebo versus 903.4 IU/mL [IQR 409.8–1701.9] in BCG, respectively [p = 0.30 for both]) (Figure 4C). In those aged 70–79 years (Figure 4D) and 80+ years (Figure 4E), BNT162b2 was mostly administered but antibody responses were not different between BCG and placebo (median 679.5 IU/mL [IQR 350.2–1213.6] in placebo versus 690.2 IU/mL [IQR 309.4–1276.7] in BCG [p = 0.61] and median 165.3 IU/mL [IQR 59.4–437.6] in placebo versus 248.5 IU/mL [IQR 145.7–405.2] in BCG [p = 0.34], respectively).

### Heterogeneity in serological response after COVID-19 vaccination in immunocompetent individuals

Those vaccinated with mRNA vaccines showed a higher concentration of circulating antibodies than those vaccinated with a viral vector vaccine (Figure 2A). Nineteen of the participants (2%), who were not known with an immunodeficiency and did not use immune suppressive medication, had negligible IgG RBD antibody concentrations (<25 IU/mL) after full vaccination. On the other side, 6 (0.6%) of the analyzed subjects stood out because of extremely high circulating antibodies (>10,000 IU/mL); two of these participants even had IgG-S and IgG RBD above 20,000 IU/mL. We cannot fully exclude the possibility of unrecognized SARS-CoV-2 infection in these cases.

## Discussion

In this study, we found that recent BCG vaccination did not enhance IgG RBD antibody concentrations in people vaccinated with specific COVID-19 vaccines. Previously we have shown the increase in IgG RBD antibody concentration after SARS-CoV-2 infection and increased interleukin-6 (IL-6) production by PBMCs in response to SARS-CoV-2 in those previously vaccinated with BCG.^8^ However, the present study suggests that this finding cannot be extrapolated to vaccination-induced antibody responses in older adults. In addition, we found that antibody production after mRNA vaccination is negatively correlated to age and male sex, as previously described by various studies.^17^^,^^18^^,^^19^

The lack of effect of BCG on COVID-19 serology differs from other studies, which have shown a synergistic effect of BCG on SARS-CoV-2 serology after mRNA or viral vector vaccination or after infection.^8^^,^^15^^,^^16^ There are several potential explanations for our findings, most importantly the time between BCG and COVID-19-specific vaccination. The interval between BCG and full COVID-19 vaccination was approximately a year but highly variable. Some individuals received the SARS-CoV-2 vaccine 8 months and others 16 months after BCG vaccination. The studies in which an improved anti-SARS-CoV-2 vaccination response was found in BCG-vaccinated individuals followed a different scheme with a much shorter interval between the vaccines (around 3 weeks) compared with our study and included mainly younger people. As the time between BCG vaccination, first COVID-19 vaccine, last COVID-19 vaccine, and sample collection varied greatly, it is difficult to determine the exact effects of specific intervals. With an increased interval between BCG and first COVID-19 vaccine, there was a reduction in time between last COVID-19 vaccine and sample collection. Especially in the most recently COVID-19-vaccinated group before sample collection, although not significant, there seemed to be an increased IgG RBD concentration in those who received a BCG vaccine. A possible explanation could be that BCG vaccination affects the maximum antibody production, whereas this change is not detectable anymore with the waning of antibodies; however, with the current data this effect cannot definitively be determined.

For most of the analyzed population, the study vaccination was the primary vaccination with BCG as the Netherlands has never included BCG in their national immunization program, in contrast to most other countries worldwide. Only roughly 25% of those included in the RCT self-reported having been previously vaccinated with BCG. Most of these individuals were vaccinated as part of occupational health programs as young adults, e.g., healthcare personnel until 1979. Other studies were done in countries that did have universal BCG coverage for infants, meaning their studies were revaccination studies.

We identified an improved antibody response in older women compared with older men. Dimorphism between the sexes is described for humoral responses after vaccination, and many underlying mechanisms may play a role,^20^ although what definitively causes the dimorphism remains elusive, especially in older populations. Some differences are attributed to hormonal influences; however, this probably plays a smaller role in post-menopausal women.^21^ Another explanation is the differential expression of immune genes, as many immune genes are X-linked. An example is the gene for TLR7 that is on the X chromosome and displays a dose-dependent response in men and women and influences susceptibility to COVID-19 infection.^22^^,^^23^ As TLR7 is important for the recognition of viruses, these differences may provide a partial explanation for the improved antibody response in women. For all age categories it is therefore important to consider these differences in making vaccination policies; it has previously been suggested to reduce vaccine doses for women in order to reduce side effects, while still being effective.^20^^,^^24^ Also, more or more immunogenic vaccines in men and older adults could be considered as they usually have poorer outcomes of infectious diseases and a reduced antibody response after vaccination.

To conclude, no significant differences in IgG RBD antibody concentration after COVID-19 vaccination were found between older adults who had been BCG-vaccinated in the year preceding COVID-19-specific vaccination compared with those who received a placebo vaccine.

### Limitations of the study

There are also limitations to this study. We only assessed the concentration of circulating antibodies against spike protein, nucleocapsid protein, and the spike receptor-binding domain. Even though antibody responses against RBD are considered a good indicator of neutralizing ability, levels alone are no definite proof, as no protective cut-off value has been agreed on as correlate of protection. IgG RBD and IgG S-protein are strongly correlated with a Spearman correlation coefficient of 0.96. (p < 0.001). In addition, we chose not to report N-protein results even though they are commonly referred to for determining undiagnosed COVID-19. In pre-COVID era samples there was a range from 0.3 to 124 IU/mL. When removing individuals from our dataset who have an IgG N-protein that falls outside of this pre-COVID era range, it does not affect our conclusion (n = 2, data not presented). With a baseline IgG N-protein we might have been able to detect changes and therefore possible missed COVID-19 diagnoses. The primary study was randomized, but the current analysis was not a fully randomized population. What stands out at baseline is the imbalance between the sexes, with relatively more women in the BCG-vaccinated group. We know men are more likely to contract severe COVID-19 and thus, with more symptoms, may have been more likely to get tested more for COVID-19. Therefore, we cannot exclude the possibility that there may have been relatively more missed COVID-19 diagnoses in the BCG-vaccinated group and in the group of women.

Due to the low number of participants having received the Ad26.COV.S and mRNA-1273 vaccine, we were not able to study these vaccines specifically. However, it is likely that results for these vaccines will be similar to ChAdOx1-S/nCoV-19 and BNT162b2, respectively, as they make use of similar vaccine platforms and vaccine antigen, although the number of participants vaccinated with ChAdOx1-S/nCoV-19 was also small and its statistical power may be limited.

## STAR★Methods

### Key resources table

**Table:** 

REAGENT or RESOURCE	SOURCE	IDENTIFIER
**Antibodies**		

Phycoerythrin-conjugated goat anti-human IgG	Jackson Immunoresearch	Cat# 109-116-170; RRID: AB_2337681
First International Standard for anti-SARS-CoV-2 immunoglobulin, human	NIBSC	NIBSC# 20/136

**Biological samples**		

Human adult EDTA plasma from venous blood	This study	N/A
Human adult serum from capillary blood	This study	N/A

**Chemicals, peptides, and recombinant proteins**		

Stabilized Trimeric Spike Protein SARS-CoV-2 (D614G mutant)	Excellgene SA	N/A
Receptor Binding Domain SARS-CoV-2, monomeric	Excellgene SA	N/A
Nucleocapsid-His Recombinant Protein	Sino Biologicals	40588-V08B

**Software and algorithms**		

Graphpad Prism 8.3.0	Graphpad software	https://www.graphpad.com/
Research Follow App	Your Research	https://yourresearch.com/
Bio-Plex Manager 6.2	Bio-Rad	Bio-Plex Manager Software, Standard Edition | Bio-Rad

**Other**		

FlexMap3D System	Luminex	https://www.luminexcorp.com/flexmap-3d-system/

### Resource availability

#### Lead contact

Further information and requests should be directed to and will be fulfilled by the lead contact, Esther J.M. Taks (Esther.Taks@radboudumc.nl).

#### Materials availability

This study did not generate new unique reagents.

### Experimental model and study participant details

#### Ethical committee approval

The study was approved by the Arnhem-Nijmegen medical ethical committee (NL73430.091.20), and registered in the EU Clinical Trials Register (2020-001591-15) and ClinicalTrials.gov (NCT04417335). All participants provided written informed consent and the trial was conducted according to the Declaration of Helsinki principles and the Guidelines for Good Clinical Practice. The study was monitored by an independent data and safety monitoring board.

#### Participant characteristics

2014 immunocompetent older adults aged 60 years or older were included in this trial. At inclusion the youngest participant was 60 years old and the oldest 93. Participant information on sex and age were self-reported at inclusion and reported in Table 1 and in Table S1 for those not included in this analysis. BCG vaccination prior to the study was not an exclusion criteria, but was self-reported at inclusion. The randomisation of participants was performed using a dynamic computer-generated algorithm, and was stratified according to age category at inclusion and inclusion site. Information on gender, ethnicity and socioeconomic status was not collected.

### Method details

#### Trial procedures

The analyses described in this paper were part of the BCG-CORONA-ELDERLY trial, a multicentre randomised placebo-controlled trial which took place between April 2020 and September 2021 in the Netherlands.^8^ The participants received either 0.1 mL 0.9% NaCl or BCG (Danish strain 1331, SSI, Denmark) intradermally and were followed for one year using digital health questionnaires (ResearchFollowApp, Your Research, the Netherlands).^25^ The trial took place in the Radboud university medical center, Nijmegen, and University Medical Center Utrecht, Utrecht, both located in the Netherlands. All participants received BCG or a placebo injection between the 16^th^ of April and the 15^th^ of May 2020. The primary endpoint was the cumulative incidence of clinically relevant respiratory tract infections including COVID-19. No differences between the treatment arms were found.^8^

#### SARS-CoV-2 vaccination

In the Netherlands, the vaccination campaign against COVID-19 started on January 6 2021 with the mRNA vaccine BNT162b2 and was shortly thereafter expanded with mRNA-1273 vaccination.^26^ For those aged 60–70 years old viral vector ChAdOx1-S/nCoV-19 vaccination was also available. Although a correlate of protection has not yet been identified to determine an adequate immune response, antibodies against the Spike protein are generally considered protective when assessing SARS-CoV-2 vaccines.^27^^,^^28^

Vaccination status was defined according to the Dutch government in place at that time.^29^ Fully vaccinated was defined as having received 2 vaccinations with BNT162b2, mRNA-1273, or ChAdOx1-S/nCoV-19 at least 14 days before blood sampling, or 1 vaccination with Ad26.COV.S at least 14 days prior to blood sampling. Any of the SARS-CoV-2 vaccinations in combination with a positive SARS-CoV-2 PCR test in the 6 months before vaccination were also considered fully vaccinated by the RIVM. However, since we are assessing the effect of vaccination, we removed these participants from the analyzed population. Vaccination history was self-reported by individuals in the end questionnaire of the study, participants were also asked to report their status when sending their serum samples.

#### Antibody testing

EDTA plasma samples from participants before COVID-19 vaccination were collected in March 2021, approximately 11 months after BCG/placebo injection. Serum samples post COVID-19 vaccination were collected at the end of the study between June and August 2021. All participants still actively participating in the study were given the option to do an antibody test. If they agreed, they were sent a capillary blood collection kit for ‘at home’ use and collected 300μL blood in a serum tube. The samples were mailed to the participating centers on the day of collection. Upon arrival at the lab, which could be up to 3 days after collection by the participant, samples were centrifuged for 10 min at 1200g and serum was stored at −20°C. Participants provided additional information on COVID-19 symptoms, vaccination and SARS-CoV-2 tests in the period between final diary entry and blood collection.

A SARS-CoV-2 fluorescent-microsphere-based multiplex immunoassay was performed, measuring Immunoglobulin G (IgG) against Spike protein, IgG against Nucleocapsid protein, and IgG against the receptor binding domain on the Spike protein (RBD).^30^^,^^31^ The samples were diluted in SM01/1% BSA and incubated with microspheres for 45 min at room temperature. After incubation the fluorescent microspheres were washed three times with PBS/0.05% Tween 20 and incubated with phycoerythrin-conjugated goat anti-human IgG (Jackson Immunoresearch, 109-116-170) for 20 min. After this final incubation step the samples were washed three times again and run on the Luminex FlexMap3D System. Using the First International Standard for anti-SARS-CoV-2 immunoglobulin, human (NIBSC, 20/136), standard curves were created. Mean Fluorescence Index (MFI) was then converted to International Units (IU/mL).We reported only the IgG RBD concentrations, as it is considered to be a good predictor of antibody neutralisation capacity.^32^ Concentrations of IgG RBD N-protein and S-protein according to number and of COVID-19 vaccine can be found in Table S2.

### Quantification and statistical analysis

The primary outcome for this analysis is the effect of BCG vaccination on the SARS-CoV IgG RBD antibody concentration after about 12 months of follow-up in participants fully vaccinated against COVID-19 who had not reported a natural SARS-CoV-2 infection. For a summary of SARS-CoV-2 antibody concentrations from all participants, including partially vaccinated and unvaccinated, see Table S1. Additionally, we assessed the effect of age, sex and vaccine type on the serological response irrespective of recent BCG or placebo vaccination.

Included in the analysis were all individuals who met the criteria of fully vaccinated and that had not reported a COVID-19 infection between start of the study and sample collection. Those unvaccinated (n = 19), partially vaccinated (n = 441), fully vaccinated but COVID-19 infection between start of study and sample collection (n = 60), and incomplete vaccination history (n = 90) were excluded from the analysis.

Time to vaccination curves were compared using Log rank test, the humoral immune assays were analyzed using the Mann-Whitney U-test and Spearman correlation in GraphPad Prism 8.3.0 (CA, USA). Spearman correlation coefficients are presented as r (number of observations) = correlation coefficient. Asterisks indicate statistical significance (∗, p < 0.05, ∗∗, p < 0.01, ∗∗∗, p < 0.001). N represents number of participants. For all figures statistical test and dispersion measures are reported in the figure legend.

### Additional resources

#### Trial registration

This trial was registered in the EU Clinical Trials Register (2020-001591-15) and on ClinicalTrials.gov (NCT04417335).

## Data Availability

All data reported in this paper can be made available upon reasonable request from the lead contact. This paper does not report original code. Any additional information required to reanalyse the data reported in this paper is available from the lead contact upon request.
