# Supplementary material for: The impact of Bacillus Calmette-Guérin vaccination on antibody response after COVID-19 vaccination
Source: iScience. 2023 Sep 28;26(11):108062. doi: 10.1016/j.isci.2023.108062 (PMC10583058; doi:10.1016/j.isci.2023.108062)
Supplement: Document S1. Tables S1–S3 [file mmc1.pdf]

iScience, Volume *26*

Supplemental information

The impact of Bacillus Calmette-Guérin vaccination on antibody response after COVID-19 vaccination

Esther J.M. Taks, Simone J.C.F.M. Moorlag, Konstantin Föhse, Elles Simonetti, Christa E. van der Gaast-de Jongh, Cornelis H. van Werkhoven, Marc J.M. Bonten, Jaap ten Oever, Marien I. de Jonge, Janneke H.H.M. van de Wijgert, and Mihai G. Netea

Table S1: Baseline characteristics of participants not included in analysis, Related to Table 1

Variable	Placebo n=552 (54.9%)	BCG n=517 (51.3%)
**Median age (IQR)- yr***	66 (62-70)	66 (63-70)
**Age category – no. (%)***		
**60-69**	408 (73.9)	384 (74.3)
**70-79**	113 (20.5)	114 (22.1)
**80+**	31 (5.6)	19 (3.7)
**Sex – no. (%)**		
**Male sex**	297 (53.8)	283 (54.9)
**Female sex**	255 (46.2)	234 (45.3)
**Median BMI (IQR) – kg/m2**	25.2 (23.3-27.5)	25.2 (23.2-27.7)
**Comorbidities – no. (%)**		
**Diabetes mellitus**	37 (6.7)	40 (7.7)
**Hypertension**	162 (29.3)	148 (28.6)
**Cardiovascular disease**	101 (18.3)	85 (16.4)
**Chronic kidney disease**	13 (2.4)	7 (1.4)
**Asthma**	38 (6.9)	29 (5.6)

BCG-Bacillus Calmette Guérin
*Age at baseline

Table S2: IgG RBD concentration in relation to interval between BCG and first COVID-19 vaccine, Related to Figure 2d

	**Placebo (n=454)**			**BCG (n=491)**			
Interval in months (days)	n	Median interval last vaccine and sample (IQR), days	Median IgG RBD (IQR), IU/mL	n	Median interval last vaccine and sample (IQR), days	Median IgG RBD (IQR), IU/mL	p-value
8 (240-269)	9	131 (126.5-149.5)	110.1 (68.01-246.3)	12	132.5 (124-141.3)	144.7 (104.6-199.2)	0.65
9 (270-299)	30	100.5 (84.3-113.3)	241.6 (100-717.4)	44	98.5 (90.3-108)	248.9 (117.7-463.1)	0.63
10 (300-329)	55	42 (20-75)	301.9 (120.7-614.9)	70	42 (25-71)	341 (186.7-664.3)	0.55
11 (330-359)	252	35 (26-46)	619.9 (294-1194)	245	36 (26-45.5)	668.8 (258.1-1257)	0.85
12 (360-389)	107	27 (19-32)	843.1 (431.4-1735)	120	25 (20.3-31)	1219 (628.6-2036)	0.09
13 (390-419)	1	15	0.8	0	n.a.	n.a.	n.a.

BCG- Bacillus Calmette Guérin RBD – Receptor binding domain

Table S3: Antibody concentrations by vaccine type and number, Related to Figure 2

	Placebo				BCG			
Vaccine	n	IgG S-protein (IU/mL)	IgG N-protein (IU/mL)	IgG RBD (IU/mL)	n	IgG S-protein (IU/mL)	IgG N-protein (IU/mL)	IgG RBD (IU/mL)
**No confirmed SARS-CoV-2 infection**								
**ChadOx1-S/nCoV-19**								
1	62	57.8 (31.3-146.3)	0.8 (0.8-2.0)	28.6 (0.8-75.5)	53	67.3 (33.7-134.3)	0.8 (0.8-1.9)	25.2 (0.8-74.8)
2	123[#]	167.1 (85.6-410)	1.2 (0.8-3.3)	119.4 (54.2-319.4)	123	175.8 (90.1-457.3)	0.8 (0.8-3.3)	134.2 (49.9-336.8)
**BNT162B2**								
1	19	329.2 (107.3-1168.9)	0.8 (0.8-5.3)	263.6 (59.0-875.0)	21	133.0 (62.2-578.2)	0.8 (0.8-1.1)	64.6 (21.6-554.4)
2	449	865.6 (435.0-1631.5)	0.8 (0.8-2.2)	685.0 (281.10-1381.5)	507	930.8 (416.3-1747.4)	0.8 (0.8-2.2)	708.0 (271.4-1388.1)
**mRNA1273**								
1	0	n.a.	n.a.	n.a.	0	n.a.	n.a.	n.a.
2	6	1800.8 (888.6-2783.0)	0.8 (0.8-0.8)	1162.9 (689.8-2328.0)	8	1953.3 (649.1-2676.0)	0.8 (0.8-0.8)	1255.8 (250.5-2634)
**Ad26.COV.S**								
1	0	n.a.	n.a.	n.a.	2	0.9-66.4	0.8-28.4	0.8-32.0
**No vaccine**								
	6	0.9 (0.9-0.9)	2.2 (1.2-12.1)	0.8 (0.8-2.0)	12	0.9 (0.9-2.8)	0.8 (0.8-2.0)	0.8 (0.8-0.8)
**PCR-confirmed SARS-CoV-2 infection before COVID-19 vaccination**								
**ChadOx1-S/nCoV-19**								
1	8	1547.1 (1001.3-2285.2)	87.9 (12.3-212.0)	1485.8 (1137.8-2003.1)	3	1770.8 (1647.0-1933.3)	61.9 (47.7-265.0)	1487.3 (1398.6-1524.2)
2	4	814.3 (601.5-1487.4)	20.9 (4.9-30.0)	647.0 (411.2-1202.4)	5	1134.0 (507.4-1725.7)	66.7 (37.3-107.6)	887.4 (434.3-1262.5)
**BNT162B2**								
1	8	2378.2 (672.7-6990.8)	10.6 (3.7-132.0)	1352.0 (294.5-3491.4)	11	3077.8 (2408.2-4695.9)	22.9 (6.3-66.6)	2119.4 (1550.0-2674.7)
2	13	3890.1 (1424.0-5289.2)	9.2 (2.8-15.4)	2953.5 (1351.3-4677.7)	17	2042.9 (971.8-4086.1)	12.3 (1.5-53.0)	1769.2 (823.0-3573.4)
**mRNA1273**								
1	0	n.a.	n.a.	n.a.	0	n.a.	n.a.	n.a.
2	0	n.a.	n.a.	n.a.	1	474.1	0.8	524.6
**Ad26.COV.S**								
1	0	n.a.	n.a.	n.a.	0	n.a.	n.a.	n.a.
**No vaccine**								
	1	76.1	18.95	54.91	0	n.a.	n.a.	n.a.

BCG-Bacillus Calmette Guérin RBD-Receptor binding domain PCR-polymerase chain reaction

Data irrespective of meeting the definition of fully vaccinated individuals, presented as median (IQR)

[#]2 participants received both the ChAdOx1-S/nCoV-19 vaccine and BNT162B2, 1 participant reported having received the ChAdOx1-S/nCoV-9 and Ad26.COV.S. These 3 individuals have not been included in this table
